# Percutaneous endovascular biopsy for the diagnosis of intravascular masses: a single-center retrospective study

**DOI:** 10.3389/fmed.2026.1950522

**Published:** 2026-09-23

**Authors:** ShuWen Ye, ShiHao Zhao, Zhen Li

**Affiliations:** Department of Interventional Radiology, The First Affiliated Hospital of Zhengzhou University, Zhengzhou, Henan, China

**Keywords:** angiography, early diagnosis, endovascular biopsy, interventional radiology, intravascular mass, pathology

## Abstract

**Background/objectives:**

Intravascular masses remain difficult to characterize with imaging alone, and histopathological confirmation is often required to guide individualized treatment. This study aimed to evaluate the technical success, diagnostic yield, and procedural safety of percutaneous endovascular biopsy (PEB) for the pathological diagnosis of intravascular masses.

**Methods:**

We retrospectively reviewed 30 patients (18 men and 12 women; mean age, 55 ± 10 years) who underwent PEB for pathological diagnosis between January 2016 and December 2023 at a single center. Tissue samples were obtained using 7-F biopsy forceps under digital subtraction angiography (DSA) guidance. Technical success, diagnostic yield, and procedure-related complications were analyzed.

**Results:**

Technical success was achieved in all patients (100%; 95% CI 88.6%–100%), with a median of three samples (range 3–4) obtained per lesion. No procedure-related complications were observed in this cohort. Histopathological analysis provided a definitive diagnosis in all cases (100%; 95% CI 88.6%–100%). Malignant lesions were identified in 11 patients (36.7%; 95% CI 21.9–54.5%), with hepatocellular carcinoma with vascular invasion being the most common malignant diagnosis. Benign lesions accounted for 19 cases (63.3%; 95% CI 45.5–78.1%) and consisted mainly of fibrous tissue and thrombi.

**Conclusions:**

PEB achieved high technical success and diagnostic yield, with no observed procedure-related complications in this selected single-center cohort. These findings suggest that PEB may be a useful diagnostic tool when imaging findings are inconclusive. Larger prospective multicenter studies are required to establish its diagnostic accuracy and clinical utility.

## 1 Introduction

Pathological biopsy remains a cornerstone of disease diagnosis and is widely used across clinical disciplines. Common approaches include open surgical biopsy, image-guided percutaneous biopsy, endoscopic biopsy, and biopsy of non-vascular luminal lesions under digital subtraction angiography (DSA) guidance (1). Primary tumors of the heart and major vessels are rare. These malignancies, which are often sarcomatous, are associated with poor outcomes because they are uncommon and frequently diagnosed at an advanced stage (2, 3). Although open surgical biopsy may provide diagnostic tissue, its invasiveness limits its use in many patients. Percutaneous needle biopsy is widely used for solid tumors, whereas endoscopic biopsy is preferred for lesions involving non-vascular luminal organs such as the esophagus, gastrointestinal tract, trachea, and ureter (4). Vascular luminal obstruction may be caused by benign or malignant disease; however, accurate differentiation is often challenging (2–5). Conventional percutaneous biopsy may be technically difficult and associated with a substantial bleeding risk when lesions are located deep within major vessels. Without histopathological confirmation, the development of precise and individualized treatment strategies may be compromised. Percutaneous endovascular biopsy (PEB) is a minimally invasive technique in which the lesion is approached through the vascular lumen via an introducer sheath. Biopsy forceps are then used to obtain tissue directly from the intravascular mass for pathological diagnosis. The purpose of this study was to evaluate the technical success, diagnostic yield, and procedural safety of PEB and to summarize our single-center experience with this technique.

## 2 Materials and methods

### 2.1 Study design and population

This retrospective single-center study was conducted in accordance with the Declaration of Helsinki and approved by the institutional review board. All patients provided written informed consent for the interventional procedure. In view of the retrospective study design, the institutional review board waived the requirement for informed consent for study participation. The medical records of all patients who underwent PEB between January 2016 and December 2023 were retrospectively reviewed. The inclusion and exclusion criteria are as follows:

#### Inclusion criteria

Intravascular space-occupying lesion confirmed by imaging; requires histopathological guidance for treatment or definitive diagnosis; percutaneous access is deemed feasible based on imaging and vascular anatomical assessment; patient consent is obtained.

#### Exclusion criteria

Uncorrectable severe coagulation disorders; active bleeding; severe cardiopulmonary intolerance; vascular anatomy preventing safe device placement; estimated sampling may cause uncontrolled embolism in the absence of protective measures.

The study included 30 patients, comprising 18 men and 12 women, with a mean age of 55 ± 10 years. The intravascular masses were located in the superior vena cava (*n* = 3), inferior vena cava (*n* = 17), pulmonary artery (*n* = 1), portal vein (*n* = 3), and left common iliac vein (*n* = 1). One patient had a mass within a left superficial femoral artery stent, and four patients had masses within a transjugular intrahepatic portosystemic shunt (TIPS). Before PEB, all patients underwent non-invasive imaging, including computed tomography (CT; *n* = 27), magnetic resonance imaging (MRI; *n* = 2), and positron emission tomography (PET/CT; *n* = 1). The indication for PEB was clinical suspicion of malignancy or the need to obtain high-quality tissue samples for histopathological evaluation to guide treatment or establish a pathological diagnosis. The institutional review board approved the study (approval number: 2024-KY-1991-003).

### 2.2 PEB procedure

All interventions were performed in an angiography suite equipped with digital subtraction angiography. Local anesthesia was administered before venous or arterial access was obtained. Vascular access was most commonly obtained through the common femoral vein (*n* = 19) or internal jugular vein (*n* = 10). The common femoral artery was used in one patient. In venous cases (*n* = 29), 65-cm or 80-cm 8-F to 12-F vascular introducer sheaths (Cordis, USA) were used. In this cohort, there were 6 cases of 8F sheaths, 23 cases of 10F sheaths, and 1 case of 12F sheaths. Intravascular obstructive lesions were confirmed by sheath angiography before biopsy. In the single arterial case, an 8-F crossover sheath (Cook, USA) was used. For venous obstructive lesions, selective catheterization was followed by exchange over a 0.035-inch stiff guidewire (Amplatz Super Stiff, Boston Scientific, Heredia, Costa Rica). Under fluoroscopic guidance, the sheath tip was carefully positioned proximal to the mass, usually over a 0.035-inch hydrophilic guidewire. A 7-F biopsy forceps device (2.2 mm × 50 cm, 7-F Maxi-Curved; Argon Medical Devices) with stainless steel hinged jaws was advanced cautiously in the closed position. Under fluoroscopic guidance, the biopsy device was advanced beyond the tip of the introducer sheath with the forceps open and gently directed toward the mass. When resistance was encountered, tissue was grasped with the forceps and withdrawn. This process was repeated until at least three specimens had been obtained. After the biopsy, a completion angiogram was performed to exclude vascular injury. A 10-F introducer sheath was preferred when possible because it allowed continuous aspiration during biopsy with a 50-mL syringe to reduce the risk of distal embolization. During the procedure, 2,500–5,000 units (50 U/Kg) of heparin were administered intravenously. At the end of the procedure, manual compression was applied to the vascular puncture site for 5–10 min to achieve hemostasis.

### 2.3 Histopathological assessment

Specimens were fixed in 10% neutral-buffered formalin and sent to the Department of Pathology for routine processing. After paraffin embedding, tissue sections were stained with hematoxylin and eosin (H&E). In accordance with routine institutional diagnostic standards, a specimen was considered adequate when it contained representative tissue fragments with preserved architecture and sufficient cellularity to permit histopathological interpretation. All H&E-stained slides were reviewed by two pathologists at our institution, and a final consensus diagnosis was reached after joint discussion. Immunohistochemistry and special histochemical stains were not performed in this study. Histological findings were reported descriptively by the pathologists. Because immunohistochemistry and special histochemical stains were not performed, descriptive diagnoses such as fibrous tissue were not further subclassified at the pathological level

### 2.4 Outcome definitions and safety assessment

Technical success was defined as successful acquisition of at least one tissue sample during PEB. Diagnostic yield was defined as biopsy material sufficient to establish a pathological diagnosis of the intravascular mass. Histological diagnosis refers to the sampled material and does not exclude unsampled components of the mass. Clinical outcomes were assessed using institutional medical records.

The following procedure-related adverse events were systematically assessed: access-site hematoma, pseudoaneurysm, and arteriovenous fistula; bleeding requiring transfusion or additional intervention; vascular perforation or dissection; procedure-related thrombosis or vessel occlusion; distal embolization, including pulmonary embolism; infection; acute kidney injury; and death within 30 days. Events were identified by review of procedural records, hospital notes, imaging examinations, and available outpatient follow-up records from the procedure until hospital discharge and 30 days.

### 2.5 Statistical analysis

Data were analyzed descriptively; no inferential statistical tests were performed. Continuous variables are expressed as mean ± standard deviation, and categorical variables as numbers and percentages. Binomial 95% confidence intervals (CIs) for proportions were calculated using the Wilson method.

## 3 Results

### 3.1 Technical success

Technical success was achieved in all 30 patients (100%; 95% CI 88.6%–100%). A median of three specimens (range 3–4) was obtained per lesion; 25 patients (83.3%) provided three specimens and 5 patients (16.7%) provided four specimens. All specimens were considered adequate for histopathological evaluation by the reviewing pathologists.

### 3.2 Diagnostic yield

Histological evaluation of the tissue samples provided sufficient information for diagnosis in all patients. A definitive pathological diagnosis was obtained in every case in which PEB material was retrieved, corresponding to a diagnostic yield rate of 100% (95% CI 88.6%–100%).

### 3.3 Histological findings

Malignant lesions were identified in 11 patients (36.7%; 95% CI 21.9–54.5%). These included five cases of hepatocellular carcinoma with vascular invasion, one case of lung cancer metastatic to the superior vena cava, one case of testicular cancer with inferior vena cava metastasis, one case of thymic carcinoma involving the superior vena cava, one case of neuroendocrine carcinoma with superior vena cava involvement (Figure 1), one case of postoperative urothelial carcinoma with IVC involvement (case 6) and one case of clear cell carcinoma of the kidney (Figure 2, case 30).

**Figure 1:**
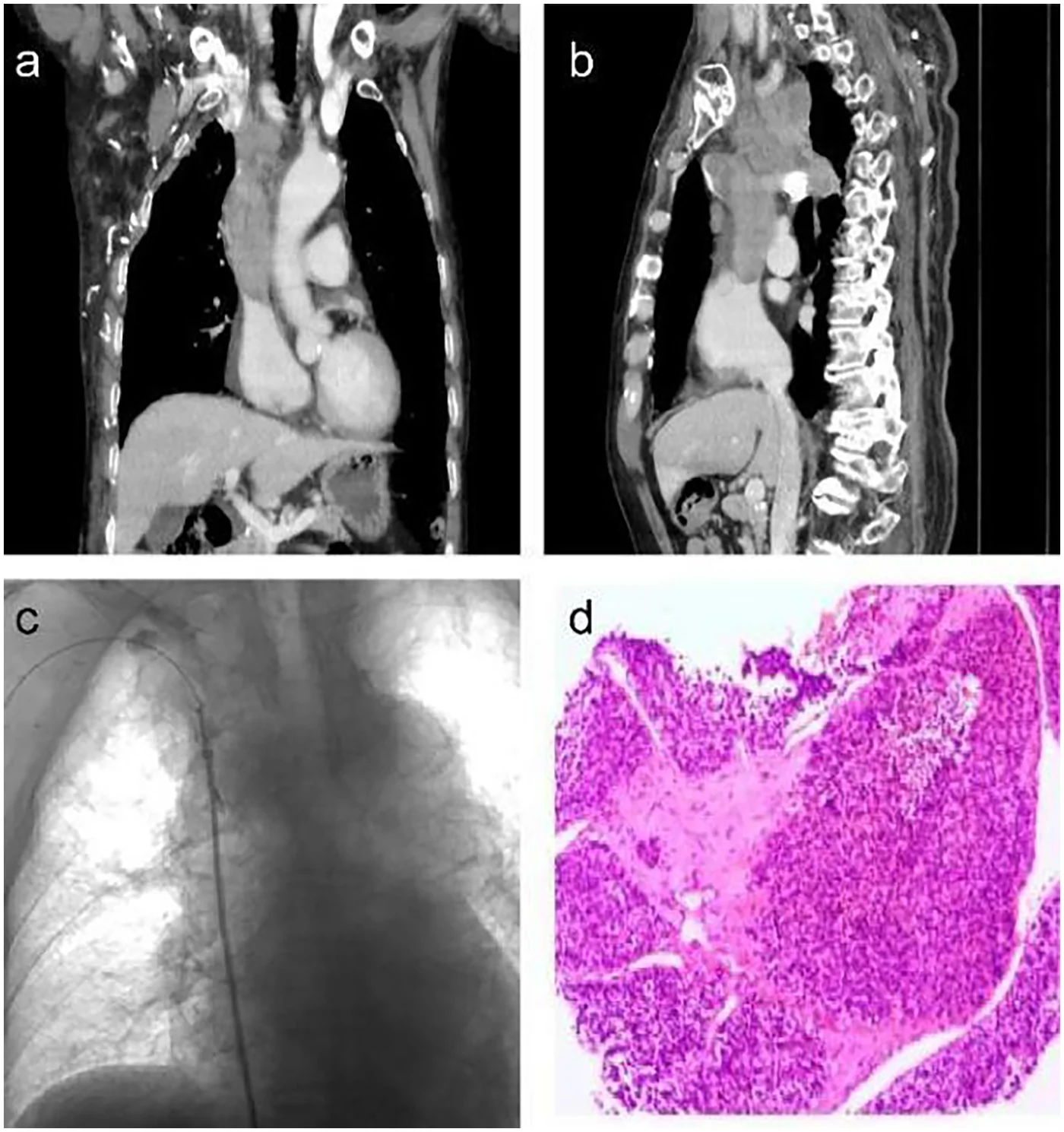
An 84-year-old man with facial edema for one week. **(a)** Coronal CT image showing an enhancing heterogeneous intravascular mass within the superior vena cava. **(b)** Sagittal CT image showing an irregularly enhancing heterogeneous mass in the superior mediastinum. **(c)** Fluoroscopic image showing placement of the introducer sheath in the superior vena cava with a 0.035-inch guidewire advanced proximal to the mass. **(d)** Histological examination confirmed neuroendocrine carcinoma (H&E,  × 200).

**Figure 2:**
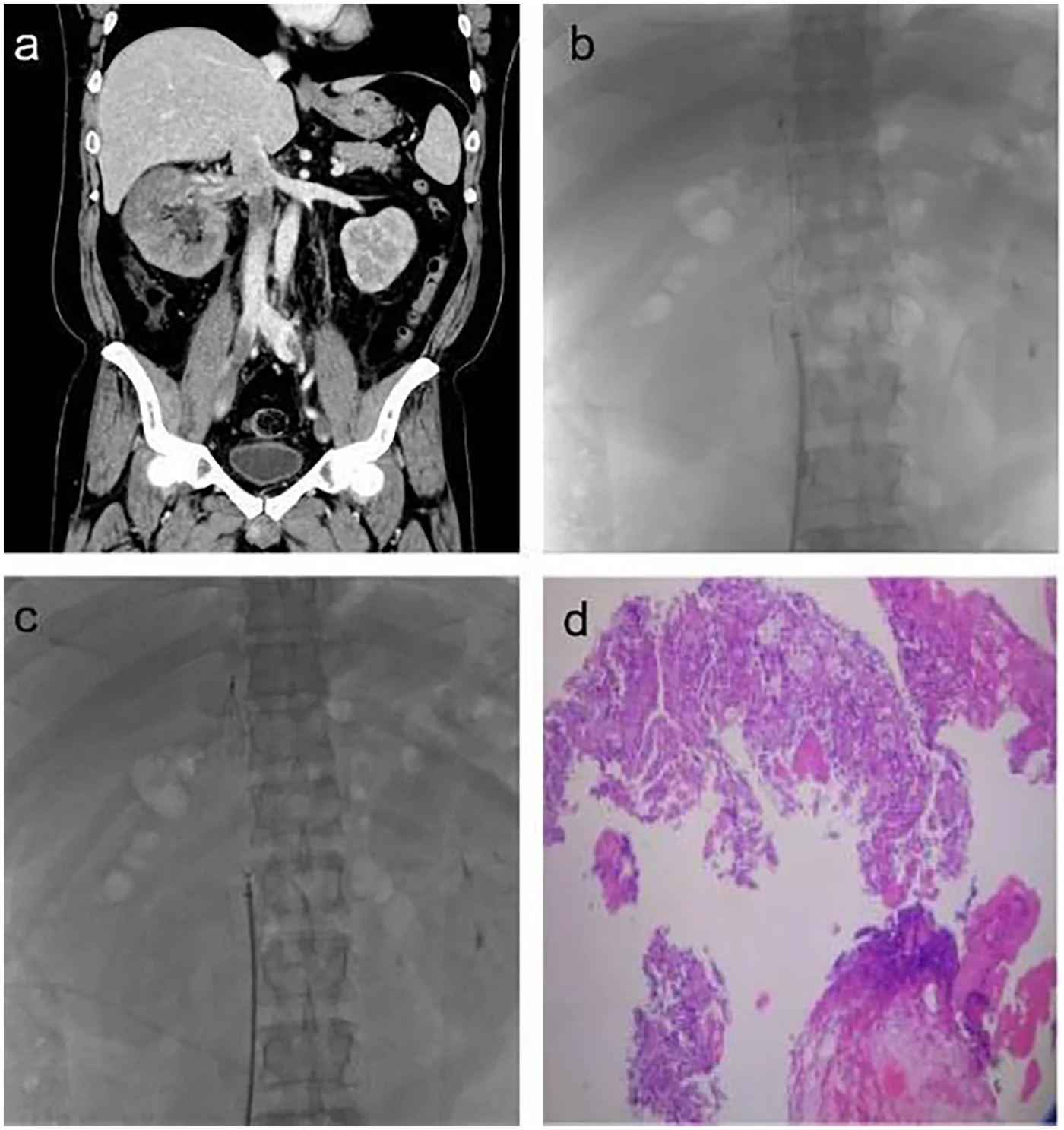
A 42-year-old man with reduced urine output and foamy urine for more than one month. **(a)** Coronal CT image showing a mass in the inferior vena cava below the right renal vein. **(b)** An inferior vena cava filter was placed before biopsy to prevent pulmonary embolism. **(c)** Under fluoroscopic guidance, the biopsy device was advanced beyond the tip of the introducer sheath and positioned against the mass. **(d)** Histological examination confirmed clear cell renal cell carcinoma (H&E,  × 200).

Benign lesions were identified in 19 patients (63.3%; 95% CI 45.5–78.1%). These included 11 cases of Budd-Chiari syndrome (cases 12–19, 21, 24, 26) (Figure 3, case 14), 4 cases of TIPS in-stent (cases 8–11) obstruction, one case of left common iliac vein thrombosis (case 5), which occurred in a patient with lymphoma. Histological examination of the sampled material showed thrombus; because only the luminal component was sampled and immunohistochemistry was not performed, lymphomatous involvement of unsampled portions of the vessel wall could not be excluded with certainty. No clinical or imaging evidence of local lymphomatous involvement was identified during 6 months of follow-up. One case of pulmonary embolism, in the single pulmonary artery case (case 28), histological examination of the forceps specimen showed thrombus, with no sarcomatous features identified in the sampled material. Because pulmonary artery intimal sarcoma may mimic thrombotic material, this diagnosis was supported by the subsequent clinical course: after AngioJet thrombectomy, follow-up imaging at 5 months showed no evidence of pulmonary artery intimal sarcoma. One case of in-stent thrombosis of the left superficial femoral artery related to lower-limb atherosclerotic disease (Figure 4, case 7), and one case of portal vein thrombosis (case 25). Fibrous tissue was reported in 14 benign lesions, comprising the four TIPS in-stent obstructions (cases 8–11) and ten Budd-Chiari syndrome lesions involving the inferior vena cava (cases 12–19, 24, 26). In the corresponding clinical contexts, these findings were consistent with chronic fibrous occlusive material: membranous/fibrous occlusion in Budd-Chiari syndrome and organised fibrous in-stent tissue in TIPS obstruction. All of these patients subsequently underwent PTA-based recanalization.

**Figure 3:**
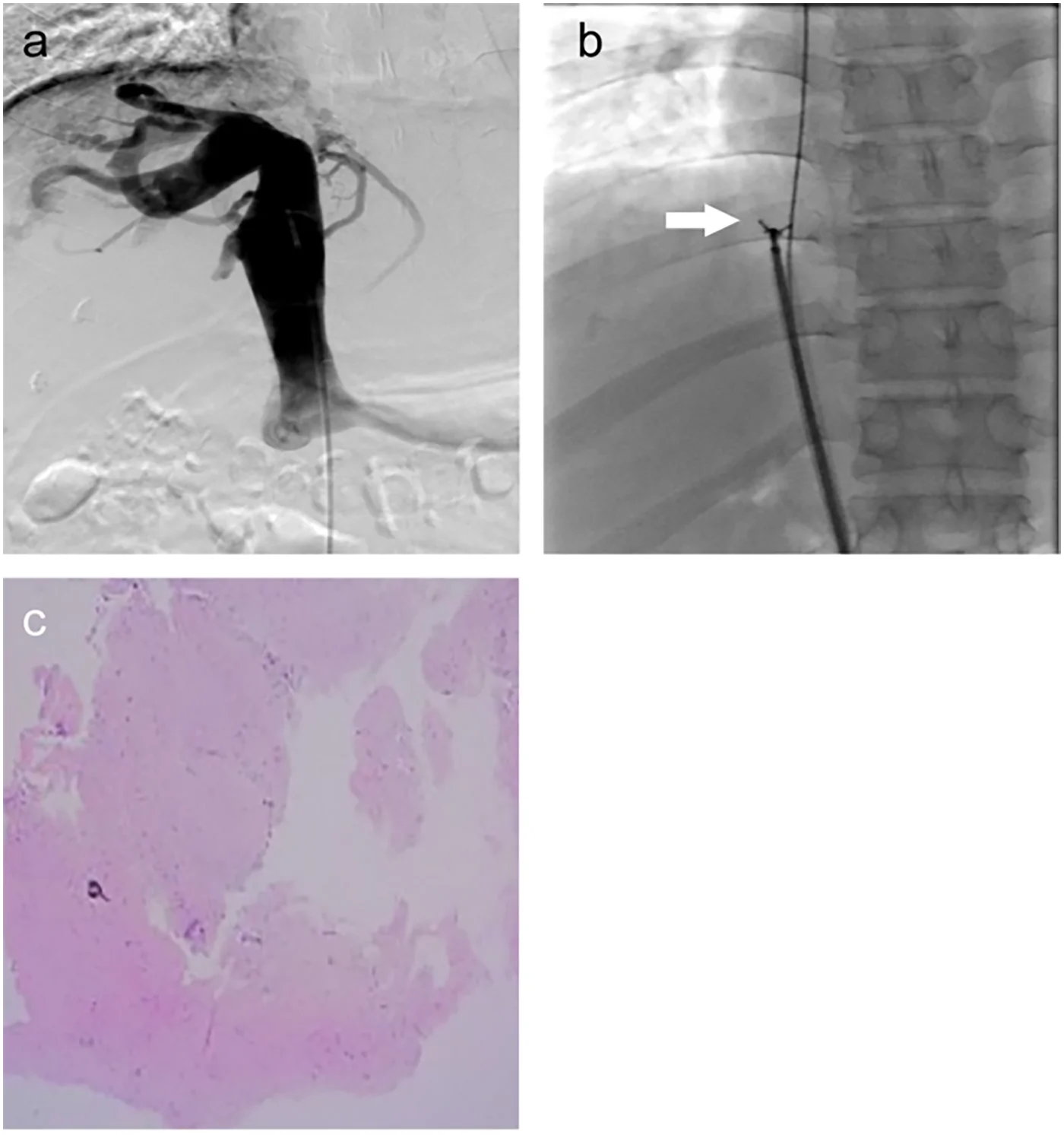
A 61-year-old man with Budd-Chiari syndrome. **(a)** Inferior vena cava angiogram showing membranous occlusion of the inferior vena cava. **(b)** Forceps biopsy of the inferior vena cava occlusion (white arrow). **(c)** Pathological examination showed fibrous tissue with inflammatory cell infiltration (H&E,  × 200).

**Figure 4:**
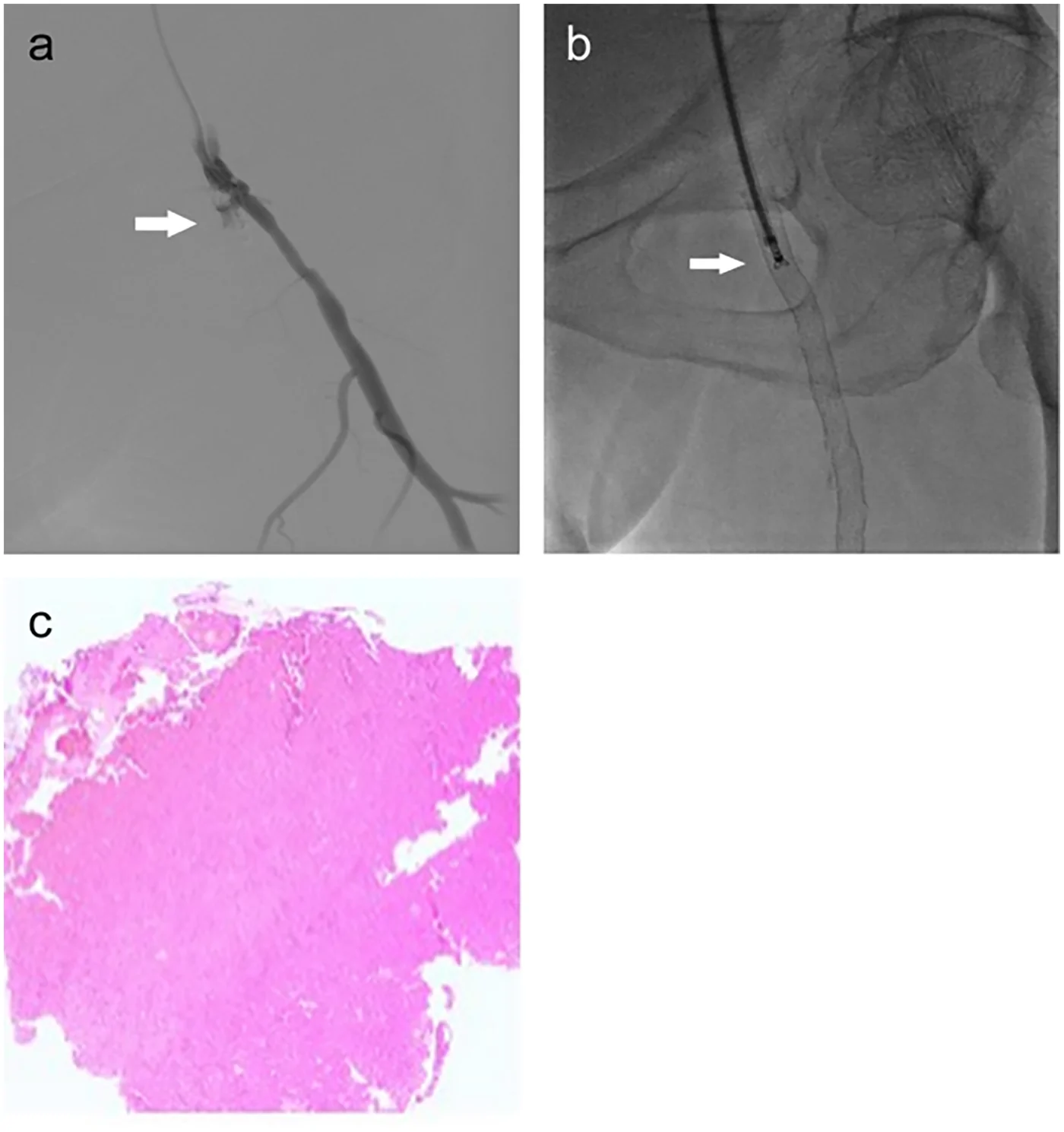
A 62-year-old man three years after stent placement for lower-limb atherosclerotic occlusive disease. **(a)** Left superficial femoral artery angiography showing in-stent occlusion (white arrow). **(b)** The introducer sheath was advanced into the stent for forceps biopsy (white arrow). **(c)** Pathological examination showed red thrombus (H&E,  × 200).

### 3.4 Follow-up

Follow-up was conducted by review of institutional medical records and outpatient visits. The median duration of last available follow-up was 6.0 months (range 3–15 months); for benign findings it was 5.5 months (range 3–15 months), and for malignant findings 7.0 months (range 5–14 months).Treatment measures administered after the pathological diagnosis were recorded for every patient. However, because pre-biopsy working diagnoses and intended treatment strategies were not systematically documented, whether the PEB result changed the presumed diagnosis or clinical management could not be reliably determined in this retrospective cohort. No benign diagnosis was revised to a malignant diagnosis during the available follow-up. Because the median follow-up in patients with benign findings was 5.5 months, longer follow-up is required to more confidently exclude delayed revision of these diagnoses.

### 3.5 Safety outcomes

Adverse events occurring within 30 days after the procedure were ascertained for all 30 patients. No procedure-related complications were observed in this cohort during the assessed period (complication rate 0/30; 95% CI 0.0–11.4%). Cases 5 and 30 involved acute thrombosis; therefore, filter implantation was performed to prevent the occurrence of pulmonary embolism, whereas filter implantation is not routinely performed in patients without acute thrombosis.

Detailed patient characteristics, imaging findings, biopsy results, and subsequent treatment are summarized in Table 1.

**Table 1:** Baseline characteristics and endovascular biopsy outcomes.

Case no.	Gender	Age	Primary disease	Imaging	Localisation	Histology	Subsequent treatment
1	M	48	Liver cancer	CT	PV	Hepatocellular carcinoma	TACE combined with targeted therapy
2	F	41	Lung cancer	CT	SVC	Large-cell lung cancer	Stent placement combined with chemotherapy
3	M	32	Testicular cancer	CT	IVC	Poorly differentiated malignant neoplasm, morphologically consistent with the patient's known testicular primary	Anti-tumour therapy
4	F	49	Thymic cancer	CT	SVC	Thymic carcinoma type B3	Endostenting combined with TACE
5	F	44	Lymphoma	CT	LCIV	Thrombus (acute thrombosis)	Filter placement and anticoagulant thrombolysis
6	M	53	Urothelial carcinoma	PET/CT	IVC	Urothelial carcinoma	Particle Radiation Therapy and immunotherapy
7	M	62	Arteriosclerosis occlusion of the lower limbs	CT	LSFA	Thrombus	Anticoagulation and thrombolysis and balloon dilatation and angioplasty
8	M	62	Cirrhosis	CT	TIPS IS	Fibrous tissue	PTA
9	F	76	Cirrhosis	CT	TIPS IS	Fibrous tissue	PTA
10	M	54	Cirrhosis	CT	TIPS IS	Fibrous tissue	PTA
11	M	62	Cirrhosis	CT	TIPS IS	Fibrous tissue	PTA
12	F	53	Budd-Chiari syndrome	CT	IVC	Fibrous tissue	PTA
13	M	56	Budd-Chiari syndrome	CT	IVC	Fibrous tissue	PTA
14	M	61	Budd-Chiari syndrome	MRI	IVC	Fibrous tissue	PTA
15	F	50	Budd-Chiari syndrome	CT	IVC	Fibrous tissue	PTA
16	M	53	Budd-Chiari syndrome	CT	IVC	Fibrous tissue	PTA
17	F	61	Budd-Chiari syndrome	CT	IVC	Fibrous tissue	PTA
18	F	55	Budd-Chiari syndrome	CT	IVC	Fibrous tissue	PTA
19	F	54	Budd-Chiari syndrome	CT	IVC	Fibrous tissue	PTA
20	M	61	Liver cancer	CT	IVC	Hepatocellular carcinoma	TACE combined with targeted therapy
21	M	49	Budd-Chiari syndrome	CT	IVC	Thrombus	PTA
22	M	84	Neuroendocrine cancer	CT	SVC	Neuroendocrine cancer	Stent placement and chemoembolisation
23	M	45	Liver cancer	MRI	IVC	Hepatocellular carcinoma	TACE combined with targeted therapy
24	F	56	Budd-Chiari syndrome	CT	IVC	Fibrous tissue	PTA
25	M	59	Portal vein thrombosis	CT	PV	Thrombus	TIPS and angiojet extraction and tube placement for thrombolysis
26	F	51	Budd-Chiari syndrome	CT	IVC	Fibrous tissue	PTA
27	M	50	Liver cancer	CT	IVC	Hepatocellular carcinoma	TACE combined with targeted therapy
28	F	65	Pulmonary embolism	CT	PA	Thrombus	Angiojet extraction
29	M	60	Liver cancer	CT	PV	Hepatocellular carcinoma	TACE combined with targeted therapy
30	M	42	Kidney cancer	CT	IVC	Clear cell carcinoma of the kidney with acute thrombosis	Filter placement and anticoagulant thrombolysis follow anti-tumour therapy

M, male; F, female; CT, computed tomography; MRI, magnetic resonance imaging; PET/CT, positron emission tomography/computed tomography; PV, portal vein; SVC, superior vena cava; IVC, inferior vena cava; PA, Pulmonary artery; LCIV, left common iliac vein; LSFA, left superficial femoral artery;TIPS IS, Transjugular Intrahepatic Portosystemic Shunt in-stent; PTA, percutaneous transluminal angioplasty; TACE, transcatheter arterial chemoembolization.

.

## 4 Discussion

Pathological diagnosis is widely regarded as the reference standard for clarifying the nature of many diseases (6). In current clinical practice, commonly used biopsy methods include endoscopic biopsy, image-guided percutaneous biopsy, and DSA-guided biopsy of luminal lesions. Transvascular pathological approaches reported in clinical practice include transjugular liver biopsy, transjugular renal biopsy, and percutaneous endovascular biopsy (7–9). CT and MRI can help characterize intravascular masses and differentiate neoplastic lesions from thrombotic lesions. However, in some cases, non-invasive imaging is insufficient to fully define the nature of an intravascular mass, even when advanced imaging techniques are used. Ultimately, histopathological examination remains essential for definitive diagnosis. Early and accurate diagnosis of the nature of an intravascular mass is important for prognosis and treatment planning (10, 11). Obtaining high-quality tissue specimens is critical for selecting appropriate treatment. For malignant masses, potential treatment options include surgical resection, interventional therapy, radiotherapy, systemic therapy, or combined approaches. For thrombotic lesions, thrombolysis, thrombectomy, anticoagulation, or endovascular reconstruction may be considered. Because major vessels are often located deep within the body, direct percutaneous biopsy may be technically difficult and may increase the risk of hemorrhagic complications. In our experience, PEB achieved high technical success and diagnostic yield, with no procedure-related complications observed in this selected cohort. Potential complications include access-site hematoma, pseudoaneurysm caused by inadvertent extravascular needle trajectory, bleeding adjacent to the vessel with injury to surrounding structures, and theoretical tumor embolization or seeding during intravascular manipulation (12, 13). From a technical perspective, the biopsy device is flexible and can access intravascular masses through tortuous vascular routes. The position of the biopsy device can be monitored in real time under fluoroscopy and angiographic guidance, helping to avoid off-target sampling. In anatomically complex cases or peripheral lesions, parallel guidewires may help stabilize both the introducer sheath and the PEB device. Primary vascular neoplasms are rare and mainly include smooth muscle tumors, whereas intravascular involvement more commonly reflects metastatic disease from other primary sites (3). One retrospective study reported six cases of intravascular primary tumors over a decade, all confirmed by endovascular biopsy (14). No primary intravascular tumors were identified in the present study. Endovascular biopsy of pulmonary artery filling defects is particularly challenging because intimal sarcoma can closely mimic chronic thromboembolic material (5). In the single pulmonary artery case in this series (case 28), the sampled material was reported as thrombus and the patient underwent AngioJet thrombectomy. Follow-up imaging at 5 months revealed no features suggestive of pulmonary artery intimal sarcoma. Nevertheless, because the biopsy sample represents only part of the lesion and the follow-up period was relatively short, the presence of an underlying intimal sarcoma cannot be entirely excluded, and longer imaging surveillance is warranted. Most malignant lesions in our cohort were metastatic, originating from the liver, lung, kidney, mediastinum, or testis. One notable case involved a malignant inferior vena cava mass caused by metastatic testicular cancer. Inferior vena cava metastasis from testicular cancer is uncommon, with only limited cases reported, and may be associated with a high risk of pulmonary embolism (15, 16). Because patients with testicular cancer may also develop inferior vena cava thrombosis, differentiating bland thrombus from tumor thrombus is clinically important. In this patient, immunohistochemistry was not performed; the diagnosis was therefore based on morphological features interpreted in the context of a known testicular primary. In this study, five benign in-stent masses were biopsied, including four cases of TIPS in-stent obstruction and one case of superficial femoral artery in-stent thrombosis. Characterizing in-stent obstructive material is valuable for investigating the etiology and pathology of stent restenosis (17). The arterial stent biopsy case also demonstrates the potential feasibility of this technique in selected arterial lesions. In patients with TIPS in-stent obstruction and Budd-Chiari syndrome, obtaining tissue that was morphologically fibrous, rather than fresh thrombus, supported an angioplasty-based approach in the subsequent endovascular treatment of these occlusions. However, because the pathological distinction was based on routine H&E morphology alone, we cannot confirm the precise substrate, such as organising thrombus, fibrous membrane, or in-stent neointimal tissue. An increasing number of imaging methods are being used to distinguish benign from malignant intravascular masses, including CT radiomics, dual-energy spectral CT, and multimodality imaging (18–20). Nevertheless, in complex clinical scenarios, pathological diagnosis remains indispensable.

This study has several limitations. First, it was a retrospective single-center study with a relatively small sample size (*n* = 30), and the lack of a control group prevented comparison of PEB with alternative diagnostic approaches. Second, the cohort was heterogeneous in terms of vascular territory, underlying disease, and histology, which limits the generalizability of the findings. Third, only patients who actually underwent PEB were included, and the number of patients considered for the procedure but not selected could not be reliably determined; the reported technical success rate and absence of complications may therefore reflect selection bias. In addition, all procedures were performed by experienced interventional radiologists at a single tertiary center. Fourth, this study assessed diagnostic yield rather than diagnostic accuracy. Because no independent reference standard such as surgical pathology, repeat biopsy, or long-term outcome confirmation was available, the PEB-derived diagnoses could not be externally validated. Furthermore, the forceps sample is obtained from the luminal aspect of the lesion, so a benign histological finding such as thrombus or fibrous tissue refers to the sampled material and does not exclude an underlying neoplasm in unsampled components of the mass. Fifth, histopathological evaluation was based on routine H&E morphology without immunohistochemistry or special histochemical stains. Fibrous tissue was therefore reported as a descriptive finding, and features such as the stage of thrombus organisation, recanalisation, haemosiderin deposition, and the inflammatory cell profile were not systematically evaluated. Therefore, the results may not be directly transferable to centers with lower procedural volumes. Future prospective multi-center studies with larger sample sizes and standardized protocols are warranted to validate these findings.

## 5 Conclusions

PEB achieved high technical success and diagnostic yield, with no observed procedure-related complications in this selected single-center cohort. These findings suggest that PEB may be a useful diagnostic tool for selected patients with intravascular masses. Larger prospective multicenter studies are required to establish its safety, diagnostic accuracy, and clinical utility.

## Data Availability

The original contributions presented in the study are included in the article. Further inquiries can be directed to the corresponding author.

## References

[1] Raab SS Grzybicki DM. Quality in cancer diagnosis. CA Cancer J Clin. (2010) 60:139–65. 10.3322/caac.20068 20444999

[2] Zhao M Nie P Guo Y Chen H. Pulmonary artery intimal sarcoma: a rare cause of filling defects in pulmonary arteries. Am J Med Sci. (2022) 364:655–60. 10.1016/j.amjms.2022.05.009 35588894

[3] Kevorkian J Cento DP. Leiomyosarcoma of large arteries and veins. Surgery. (1973) 73:390–400. 4687797

[4] Liu Y Yang F Chi X Zhang Y Fu J Bian W. Needle biopsy compared with surgical biopsy: pitfalls of small biopsy in histological diagnosis of IgG4-related disease. Arthritis Res Ther. (2021) 23:54. 10.1186/s13075-021-02432-y 33568210 PMC7874654

[5] Kwon BS Park JH Gwon DI Lee JS. Feasibility of endovascular catheter biopsy as a diagnostic modality for patients with pulmonary artery sarcoma. Vasc Endovascular Surg. (2018) 52:636–40. 10.1177/1538574418779470 29852817

[6] Susaki EA. Recent developments in automated diagnosis of pathological images and three-dimensional histopathology. Brain Nerve. (2019) 71:723–32. 10.11477/mf.1416201344 31289246

[7] St Julien J Reisenauer CJ Andrews JC Fleming CJ Misra S Takahashi EA. Transjugular renal biopsy bleeding risk and diagnostic yield: a systematic review. J Vasc Interv Radiol. (2020) 31:2106–12. 10.1016/j.jvir.2020.07.016 33023806 PMC11425240

[8] Kaufman CS Cretcher MR. Transjugular liver biopsy. Tech Vasc Interv Radiol. (2021) 24:100795. 10.1016/j.tvir.2021.100795 34895709

[9] Hong C Lin JL Chen HM Guo WL Li XY Wu XF. Percutaneous endovascular biopsy for the diagnosis of pulmonary artery masses: a preliminary single-center study. Pulm Circ. (2023) 13:e12234. 10.1002/pul2.12234 37168839 PMC10166081

[10] Ruf M. Early diagnosis is important. Dtsch Arztebl Int. (2021) 118:227. 10.3238/arztebl.m2021.0122 34090544 PMC8572539

[11] Hellyer P. Early cancer diagnosis reduces the cost of the disease. Br Dent J. (2022) 233:42. 10.1038/s41415-022-4458-5 35804128

[12] Arnold MJ Keung JJ McCarragher B. Interventional radiology: indications and best practices. Am Fam Physician. (2019) 99:547–56. 31038901

[13] Quan T Li X Xu H Lin Y Liu C Li D. Percutaneous endovascular biopsy in the diagnosis of venous sinus lesions: technical note. J Neurosurg. (2018) 131:462–6. 10.3171/2018.3.JNS173143 30141756

[14] Pomoni A Sotiriadis C Gay F Jouannic AM Qanadli SD. Percutaneous endovascular biopsy of intravascular masses: efficacy and safety in establishing pre-therapy diagnosis. Eur Radiol. (2018) 28:301–7. 10.1007/s00330-017-4983-9 28779399

[15] Soukane L Bouzas CM Marechal C Van Houte B Vazquez C. Multidisciplinary management of testicular cancer complicated by thrombosis of the inferior vena cava: a case report. Acta Chir Belg. (2018) 118:250–3. 10.1080/00015458.2017.1346037 28678675

[16] Masui S Onishi T Arima K Sugimura Y. Successful management of inferior vena cava thrombus complicating advanced germ cell testicular tumor with temporary inferior vena cava filter. Int J Urol. (2005) 12:513–5. 10.1111/j.1442-2042.2005.01073.x 15948757

[17] Zhang DM Chen SL. Potential mechanisms of in-stent neointimal atherosclerotic plaque formation. J Cardiovasc Pharmacol. (2021) 78:388–93. 10.1097/FJC.0000000000001059 34029271

[18] Chun SH Suh YJ Han K Park SJ Shim CY Hong G-R. Differentiation of left atrial appendage thrombus from circulatory stasis using cardiac CT radiomics in patients with valvular heart disease. Eur Radiol. (2021) 31:1130–9. 10.1007/s00330-020-07173-1 32812175

[19] Rohatgi S Howard SA Tirumani SH Ramaiya NH Krajewski KM. Multimodality imaging of tumour thrombus. Can Assoc Radiol J. (2015) 66:121–9. 10.1016/j.carj.2014.11.001 25660299

[20] Qian LJ Zhu J Zhuang ZG Xia Q Cheng YF Li JY. Differentiation of neoplastic from bland macroscopic portal vein thrombi using dual-energy spectral CT imaging: a pilot study. Eur Radiol. (2012) 22:2178–85. 10.1007/s00330-012-2477-3 22622347

